# Preparation and Characterization of Sludge-Based Magnetic Biochar by Pyrolysis for Methylene Blue Removal

**DOI:** 10.3390/nano11102473

**Published:** 2021-09-22

**Authors:** Huiping Zeng, Wei Qi, Longxue Zhai, Fanshuo Wang, Jie Zhang, Dong Li

**Affiliations:** 1 Key Laboratory of Water Quality Science and Water Environment Recovery Engineering, Civil Engineering, Beijing University of Technology, Beijing 100124, China; zenghuiping@bjut.edu.cn (H.Z.); qw0529@163.com (W.Q.); zlx1017@163.com (L.Z.); 6282031@163.com (J.Z.); 2 Beijing Institute of Architectural Design, Beijing 100124, China; wfs960808@163.com; 3 State Key Laboratory of Urban Water Resource and Environment, Harbin Institute of Technology, Harbin 150090, China

**Keywords:** iron mud, sewage sludge, magnetic biochar, methylene blue removal, pyrolysis

## Abstract

The development of low-cost adsorbent is an urgent need in the field of wastewater treatment. In this study, sludge-based magnetic biochar (SMB) was prepared by pyrolysis of sewage sludge and backwashing iron mud without any chemical agents. The samples were characterized by TGA, XRD, ICP, Organic element analysis, SEM, TEM, VSM and BET. Characterization analysis indicated that the magnetic substance in SMB was Fe_3_O_4_, and the saturation magnetization was 25.60 emu·g^−1^, after the adsorption experiment, SMB could be separated from the solution by a magnet. The batch adsorption experiment of methylene blue (MB) adsorption showed that the adsorption capacities of SMB at 298 K, 308 K and 318 K were 47.44 mg·L^−1^, 39.35 mg·L^−1^, and 25.85 mg·L^−1^, respectively. After one regeneration with hydrochloric acid, the maximum adsorption capacity of the product reached 296.52 mg·g^−1^. Besides, the adsorption kinetic described well by the pseudo-second order model revealed that the intraparticle diffusion was not just the only rate controlling step in adsorption process. This study gives a reasonable reference for the treatment of sewage sludge and backwashing iron mud. The product could be used as a low-cost adsorbent for MB removal.

## 1. Introduction

The pollution of water systems by various pollutants has attracted widespread attention for many years. The widespread use of dyes has made the pollution of water systems more serious; dyes are mainly discharged into wastewater from dyestuff manufacturing, food, cosmetics, printing and leather manufacturing industries [1]. It is estimated that during manufacturing and processing about 15% of the dyes are lost to the wastewater [2]. In addition, more than 200 tons of dyes are released from the textile industry as wastewater every year [3]. Among them, MB is a cationic dye, which is widely used for coloring of textiles [4]. MB is fairly toxic, contains a large amount of organic compounds, has a high solubility and is difficult to degrade [5]. Many methods have been applied for removing of dyes in wastewater. Some of these are flocculation, oxidation, electrolysis, biodegradation, ion-exchange, photo catalysis and adsorption [6,7]. Adsorption is one of the most common used methods in dye wastewater remediation. Generally, adsorption technology does not leave behind by-products and is exceptionally effective for removing dyes in water [8,9]. Activated carbon is a widely used adsorbent, but due to its high manufacturing and recycling costs, the price is relatively expensive. Biochar could be used as an alternative to activated carbon in some areas of wastewater treatment as adsorbent. Considering the use of biochar as adsorbent for wastewater treatment can greatly save the purchase cost of activated carbon. Thermal conversion provides a method to convert biomass into biochar. Thermal conversion methods mainly include pyrolysis, gasification and hydrothermal carbonization [10]. Among these methods, pyrolysis has a high efficiency, is reasonable cheap and the produced biochar usually has high application value [11,12]. Therefore, this method is one of the common techniques for converting organic waste into biochar. In this study, magnetic biochar was considered to be prepared by one-step pyrolysis.

In recent years, the sewage sludge production increases dramatically due to the population growth and the improvement of treatment capacity of sewage treatment plants. In addition, the sludge treatment has become one of the main problems of wastewater treatment plants, it requires a lot of work, energy and money [13]. Traditional treatment methods include filling, incineration, paving, dumping into the sea and turning it into building materials, but these methods can easily cause secondary pollution [14], how to transform the sludge waste into useful substances is a difficult point. Wastes such as crop waste and forestry residues have been considered for the preparation of biochar [15,16]. Considering the conversion of sewage sludge waste into biochar can not only reduce the cost of biochar preparation, but also provide a way for sludge disposal. In addition, the separation and recovery of adsorbents after adsorption is still a huge challenge [17], and traditional separation method usually requires centrifugation and filtration steps. These steps may lead to desorption, which in turn produces secondary contamination [18]. Magnetic composites could be controlled by the principle of magnetic separation. They could be attracted by magnetic field in the presence of it, and could be used as ordinary materials in the absence of the magnetic field [19]. Therefore, the magnetization of biochar is an effective strategy to meet the challenges of separation and recycling [18]. Transition metals and their oxides are usually introduced into biochar matrix to give them magnetism [20]. At present, most of the magnetic sources of biochar are iron-containing chemical agents [21,22,23]. The usage of chemical agents increases the cost of preparing magnetic biochar, which is unwanted. Therefore, some scholars have focused on the iron-containing wastes. For example, Yi et al. and Chen et al. used steel pickling wastewater as magnetic source to prepare magnetic biochar [24,25], but these wastes usually come from industrial production, which has the risk and trouble of toxic substances. The search for a cheap and non-toxic source of iron remains a research hot spot.

In the past twenty years, a large number of iron and manganese removal water plants have been established in Northeast China to treat groundwater with excessive iron and nganese content [26,27,28,29,30,31]. However, along the operation of the water plants, a large amount of iron mud would be produced, and the subsequent treatment of iron mud costs a lot [32,33]. This iron mud is cheap, easy to obtain, and contains no toxic substances [34]. This study creatively proposed to use the waterworks sludge of iron as the magnetic source, which does not contain the risk of heavy metal pollution, and provides a good method for the final disposal of waterworks sludge. In addition, no chemicals were added in the preparation process, which saved the preparation cost of magnetic biochar.

Therefore, the main purposes of this study are: (1) to attempt to use water work backwashing iron mud and sewage sludge to prepare an environmentally friendly and economical magnetic biochar through pyrolysis method; (2) to use various methods to characterize the properties of the obtained magnetic biochar; and (3) to evaluate the adsorption and regeneration properties of the adsorbent by testing the adsorption capacity of MB on the product.

## 2. Materials and Methods

### 2.1. Materials

All chemicals used in this work were analytical grade and were dissolved in deionized (DI) water. The backwashing iron mud was collected from groundwater treatment plants in the city of Harbin, Heilongjiang province of China. Previous studies have shown that the waterworks sludge is mainly contains Fe and a small amount of Si, Ca, K, and Mn, which account for 89%, 4.3%, 4.0% 2.4%, and 0.3% by mass, respectively [35]. The sewage sludge used in the study is from a sewage treatment plant in Beijing, and its physicochemical properties are shown as follow: The moisture content of the sewage sludge is 99.6%, no other high concentration metals are present, pH is 6.7 and the total carbohydrate is about 2450 mg·L^−1^. A previous study [36] shows that the average content of Cr, Cu, Ni, Pb, and Zn of sewage sludge from this plant is about 33.8 mg/L, 96.9 mg/L, 16.5 mg/L, 23.1 mg/L, and 733 mg/L, which are far lower than the sludge utilization standards of China, the European Union, and the United States [36].

### 2.2. Synthesis of Magnetic Biochar

The synthesis procedure was as follows: the untreated iron mud and sewage sludge were mixed in a ratio of 1:5 (mass ratio), the required volume is converted according to the solid content of these two kinds of sludge. The suspension was stirred and sonicated for 10 min with an ultrasonic instrument to make them fully mixed. The mixture was then dried in an oven at 80 °C for 6 h. The dried product was milled and sieved. The sieved product was placed in a closed container and pyrolyzed in a muffle furnace at a temperature of 600 °C for one hour. The sample was then washed with DI water for several times, and saved in a sealed container for later use (the preparation flow chart is shown in Figure S1).

In addition, above steps were repeated for sewage sludge to prepare biochar (BC) without magnetic.

### 2.3. Characterization

Surface crystalline was analyzed to identify the sample’s constituents using X-ray diffraction (XRD) (Bruker D8 Advance, Germany) with Co Kα radiation (l = 1.79026 A) operated at a 2θ range of 10~90°, and the operated voltage, current, and scanning speed were 40 kV, 40 mA, and 6°·min^−1^, respectively. X-ray photoelectron spectrometer (XPS) (Thermo escalab 250XI, USA) was used to detect surface elemental composition. The surface characteristics and morphology of the sample was characterized by scanning electron microscopy (SEM) and transmission electron microscopy (TEM). Magnetic property of samples was measured using a magnetometer (Quantum Design, USA) with versalab system. Total surface area was measured using N_2_ sorption on an ASAP 2460 analyzer and calculated using Brunner Emmet Teller (BET) method. The proportions of various elements were detected by Inductively coupled plasma emission spectrometer (ICP) and organic element analysis. In addition, Thermo Gravimetric Analyzer (TGA) was also performed.

The zero-charge point (pH_pzc_) of SMB was determined by adding 0.025 g sample into 25 mL 0.1 M KCl solution at different initial pH (2–12), and the final pH of each solution was measured 24 h later. Finally, the graph was drawn with the initial pH as the abscissa and the final pH as the ordinate.

### 2.4. Adsorption Experiments

First, sorption kinetics of MB at a constant concentration (10 mg·L^−1^) onto SMB were examined to obtain the contact time needed to achieve the adsorption equilibrium. Four hundred milligrams of magnetic biochar (0.5 g·L^−1^) was added to a polyethylene plastic bottle, which containing 800 mL MB solution (initial pH 6.6 ± 0.1). Then the bottles were shaken at 175 rpm under 25 °C in a thermostatic orbit shaker. Adsorption kinetic data were obtained by sampling at different times within 0–48 h, and each sampling volume was 15 mL. At each sampling point, the bottle was withdrawn, and the mixtures were immediately filtered through 0.45 μm pore size nylon membrane filters, and the residual MB concentration in the obtained solution was determined using a visible spectrophotometer at the wavelength of 665 nm. The data obtained were then fitted with various kinetic models.

The adsorption isotherm batch experiments were carried out in 200 mL conical flasks. In addition, each group included 50 mL MB solution (concentration between 1 mg·L^−1^ and 160 mg·L^−1^) and 25 mg adsorbent (initial pH 6.6 ± 0.1). The suspension was shaken in a thermostatic orbital shaker at a speed of 175 rpm for 36 h, this period has been determined by kinetic experiments previously to be sufficient to establish adsorption equilibrium. Then the mixtures were immediately filtered and determined by the same method. The adsorption isotherm experiments were carried out at 25 °C, 35 °C, and 45 °C, respectively. Isotherm data were simulated with various isotherm models.

### 2.5. Reproducibility and Reusability

In the regeneration experiment, SMB after adsorption was treated with 0.01 mol·L^−1^, 0.05 mol·L^−1^, 0.1 mol·L^−1^, and 0.5 mol·L^−1^ hydrochloric acid respectively. The regeneration time was set at 9 h [34]. The regenerated product was washed several times, and the adsorption effect of the product on MB was detected respectively. According to the results, the optimal regeneration concentration was selected as 0.5 mol·L^−1^. The adsorption kinetics and adsorption isotherm experiments were repeated for the SMB after one regeneration, and the experimental conditions are the same as Section 2.4 (only 25 °C was selected for isotherm experiment). The SMB was regenerated five times and the adsorption effect was determined respectively.

## 3. Results and Discussion

### 3.1. Characterization of Adsorbents

The adsorption rate is specific to the adsorbent and largely depends on the characteristics of the adsorbent [37]. In this study, the structural characteristics of the adsorbent were studied by detecting TGA, XPS, ICP, Organic element analysis, SEM, TEM, VSM, and BET.

**Figure 1:**
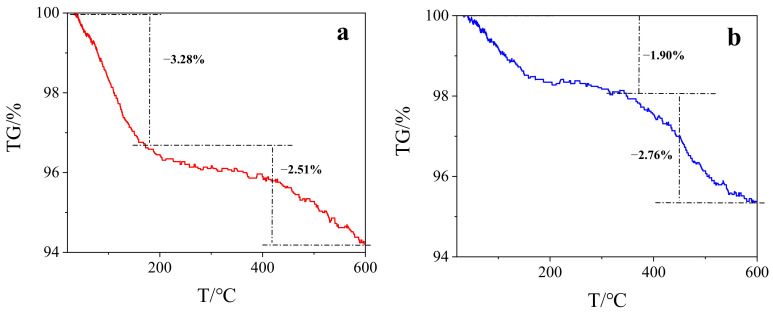
(**a**) TGA of BC; (**b**) TGA of SMB.

TGA analysis showed that both BC and SMB have the first weight loss about 100 °C, which may be caused by the evaporation of water (Shown in Figure 1). Although the sample just prepared was completely dry, it may have absorbed moisture in the air and thus contain a certain amount of water. In addition, the second weight loss occurred at about 450 °C, at which time the sample was thermally degraded. During the whole weight loss process, the mass of BC decreased by 5.79%, while the mass of SMB decreased by 4.65%. In addition, SMB was decomposed at a higher temperature, indicating that the thermal stability of the SMB was slightly higher than the original BC, which may be due to thermally inactive Fe_3_O_4_ serving as a diluent [38]. Although two weight losses occurred, the weight loss ratios of the two samples were both small, indicating that the prepared samples have a certain degree of stability. In addition, the weight loss of magnetic biochar prepared by Wang et al. was nearly 40% in TGA analysis [38], while the weight loss of SMB in this study was only 4.56%, indicating that SMB has good stability [39].

**Figure 2:**
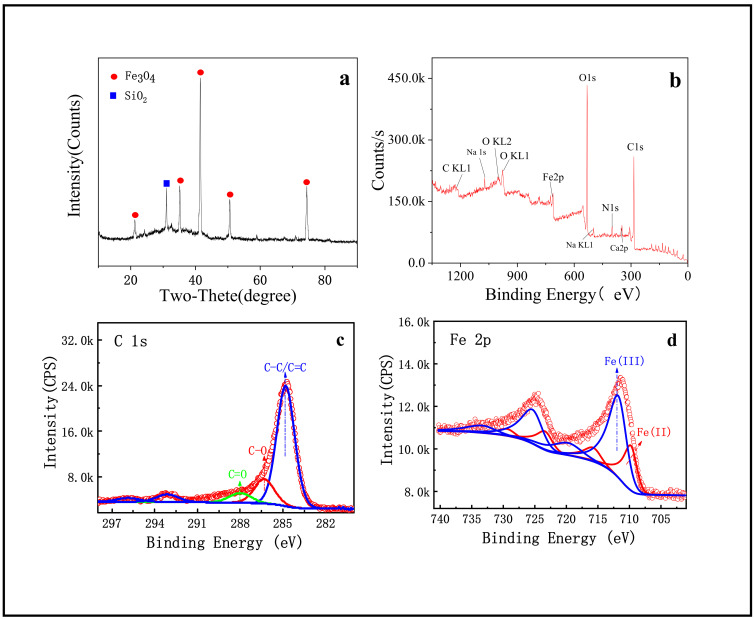
(**a**) XRD of SMB; (**b**) XPS of SMB; (**c**) C1s in high resolution spectrum of SMB; (**d**) Fe2p in high resolution spectrum of SMB.

The structure and phase purity of SMB were investigated by XRD (Figure 2a). As can be seen, the diffraction peaks marked with round shape in Figure 2a are well indexed to (111), (220), (311), (400), and (440) planes of Fe_3_O_4_ [34]. According to these strong and sharp diffraction peaks, it can be inferred that Fe_3_O_4_ with good crystallinity had been prepared. In addition, biochar is one of the main components of SMB, but no specific diffraction peaks of carbon crystal structure were found, indicating that the carbon in SMB was amorphous carbon [34]. It is not negligible that there is a strong peak belonging to SiO_2_ (square mark in Figure 2a) and some slight peaks belonging to other substances in the XRD pattern. This phenomenon may be caused by the fact that the iron mud and sewage sludge in the raw materials have not been purified and contain impurities.

It is well-known that XPS is often used to verify the elemental composition of materials. Therefore, XPS was applied for component analysis, and the full scan spectrum is presented in Figure 2b, it can be seen that the main composition elements of SMB were C, N, O, and Fe. Besides, there were some broad and low intensity peaks belonging to impurities such as Na, Ca, Si, and Al. The mass fractions of impurities in the product were all less than 5% and the concentration of these impurities can be seen in Table 1. At the same time, the strong C peak and high surface concentration (At% = 55.31) indicated that the main component of the sample surface was carbon. Therefore, the binding energy peak of C1s was displayed in the high-resolution spectrum (Figure 2c) to explore the C-containing functional groups present on the surface of SMB. It could see that the C-C/C=C peak at 284.88 ev is the strongest, and there were also C=O and C-O peaks. In addition, the high-resolution spectrum of Fe was shown in Figure 2d. The peaks of Fe^3+^ and Fe^2+^ were not equivalent, indicating that there was not only Fe_3_O_4_ in SMB but also FeOOH. FeOOH was the incompletely reacted part of the raw iron mud. The binding energy peaks of Fe was very weak, and the surface concentration was also low (At% = 4.68), indicating that the core of Fe_3_O_4_ may be hidden in the carbon shell.

Through ICP detection and organic element analysis, the element composition and proportion of BC and SMB were further analyzed (Table 1). The results showed that both materials contained lower carbon content, which is very common in sludge-based materials [40]. Elemental analysis showed that the Fe content of the SMB was 3.6-times greater than that of the BC due to the addition of waterworks sludge. The high iron content in SMB demonstrates that Fe was successfully loaded on the biochar. The mass ratio of iron in BC was 3.94%, this may be caused by the introduction of Fe in the coagulation [14] or flocculation [41] process in the sewage treatment process. However, in the experiment, it was found that the BC could not be magnetically separated from the water, indicating that the Fe introduced in the water plant’s sludge treatment process alone was not enough to make the material produce good magnetic properties. Besides, due to the dilution effects of the waterworks sludge addition, the contents of non-volatile elements such as Si, K, and Ca in the samples were reduced.

Referring to the method used by the International Biochar Initiative (IBI), the carbon storage value (BC_+100_) was used to quantify the stability of biochar. The larger BC_+100_is, the more stable biochar is. The core of this method is that when H/C mole ratio is less than or equal to 0.4, the residual percentage of organic carbon (BC_+100_) in biochar after 100 years of evolution is expected to be 70%. When H/C mole ratio is between 0.4 and 0.7, and BC_+100_ is 70%. According to a study by Huang et al., the H/C molar ratio of biochar prepared at 600 °C is between 0.13–0.76 [42]. After calculation, the H/C molar ratio of SMB was 0.42, indicating that the sample has good stability. The H/C molar ratio of BC was 0.83, and the carbon storage value of SMB was greater than that of BC, indicating that the addition of magnetic material improves the stability of BC.

**Figure 3:**
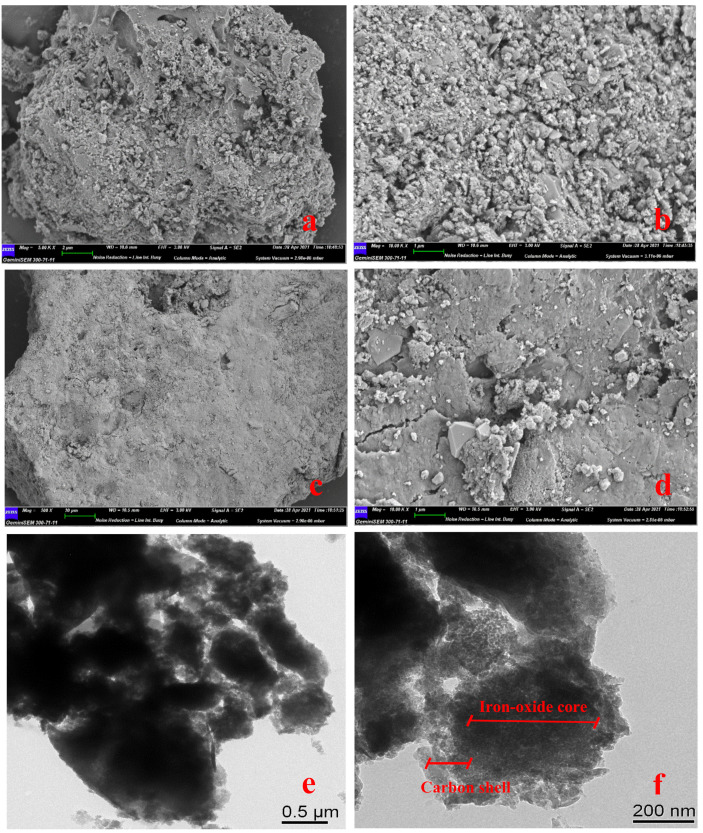
(**a**,**b**) SEM images of BC; (**c**,**d**) SEM images of SMB; (**e**,**f**) TEM images of SMB.

The details about the structure and morphology of the resulting composite material were examined in SEM and TEM images (Figure 3). Due to the nature of biochar itself, the surface of the material was rough and porous and had an irregular surface (Figure 3a,b). It could be seen that after Fe_3_O_4_ was loaded on the biochar, the surface became more smoothly, and part of the Fe_3_O_4_ particles were embedded in the biochar matrix. This indicated that a good mechanical bond was formed between the biochar matrix and the Fe_3_O_4_ particles [43]. Considering the pyrolysis process under 600 °C for one hour, there might be a minor sintering effect which activates the binder function of Fe_3_O_4_ to the biochar. Thus, the product had a certain degree of stability and would not separate under the impact of water flow. In order to further explore the internal structure and spatial distribution, the TEM was used for further characterization. Combined with XPS analysis, it was speculated that the outer gray parts were carbon shell, which covering the inner iron oxide (black part) (Figure 3f). In addition, SMB particle had irregular morphology. The nanometer measurement software was used to measure the diameter of the particles in the TEM images. The particle size of SMB ranged from 13 nm–1.45μm, indicating that the magnetic biochar produced by this work was a nano/colloidal composite material.

**Figure 4:**
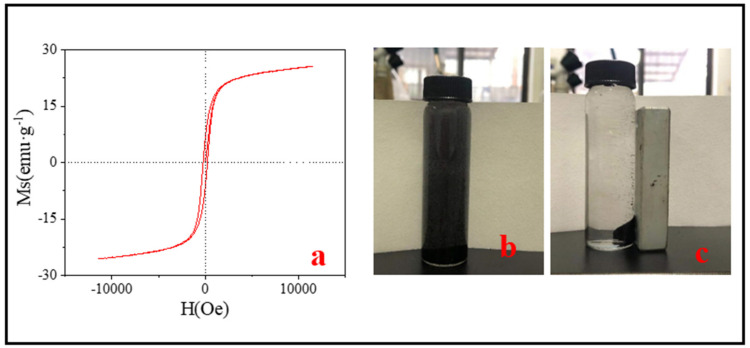
(**a**) The VSM results of SMB; (**b**) Pre-magnetic separation; (**c**) after magnetic separation.

Magnetic separation capacity is very important for the separation and recovery of adsorbents. The magnetic properties of SMB were measured by the VSM experiment. From the hysteresis loop of Figure 4, the saturation magnetization (Ms), the magnetic remanence (Mr), and the coercivity (Hc) values were 25.60 emu·g^−1^, 6.10 emu·g^−1^, and 205.20 Oe, respectively. The saturation magnetization was significantly lower than the ferromagnetism of Fe_3_O_4_ (90 emu·g^−1^) [44]. The reason for this phenomenon may be the following two aspects: (1) XRD and TEM results showed that the structure of carbon shell coated on iron oxide core possibly was formed. In addition, after measurement, the thicknesses of the carbon shells were between 16 nm–200 nm (Figure 3f). The existence of the carbon layer reduced the magnetic properties of SMB; (2) The reaction material sewage sludge and iron mud were not purified, resulting in impurities in the SMB, and the presence of these impurities had an impact on the magnetic properties of SMB. Besides, the presence of impurities may also be the reason for the presence of Mr and Hc. According to the study by Ma et al., magnetic materials can be used for magnetic separation when the magnetization reaches 16.30 emu·g^−1^ [45]. Despite the magnetic properties of SMB was only 25.60 emu·g^−1^, it could still be easily separated from the solution within 1 min with an ordinary magnet (Figure 4b,c).

**Figure 5:**
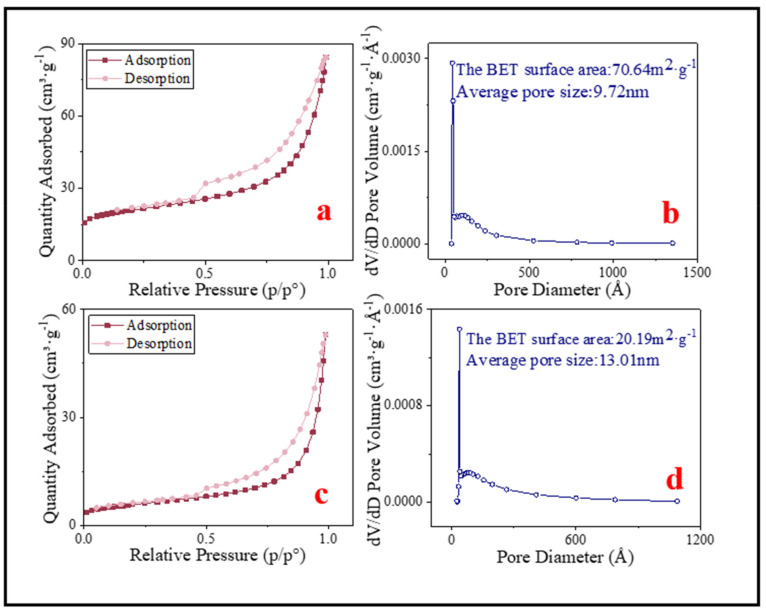
(**a**) N_2_ adsorption–desorption isotherm liner plots of BC; (**c**) N_2_ adsorption–desorption isotherm liner plots of SMB. (**b**) BJH pore size distribution of BC; (**d**) BJH pore size distribution of SMB.

According to the IUPAC classification, the N_2_ adsorption–desorption isotherms of BC and SMB both belong to type IV isotherms with hysteresis loops of type H3 (Figure 5a,c). In addition, the average pore size of the samples can be obtained from the BJH (Barrett, Joyner, and Halenda) pore size distribution chart (Figure 5b,d). All the specific surface areas and the average pore diameters were marked in Figure 5. The BET of BC and SMB were 70.64 m^2^·g^−1^ and 20.19 m^2^·g^−1^, respectively. In addition, the average pore diameters were 9.72 nm and 13.01 nm, respectively. This indicated that BC and SMB were both mesoporous materials.

### 3.2. Kinetics Modeling

**Figure 6:**
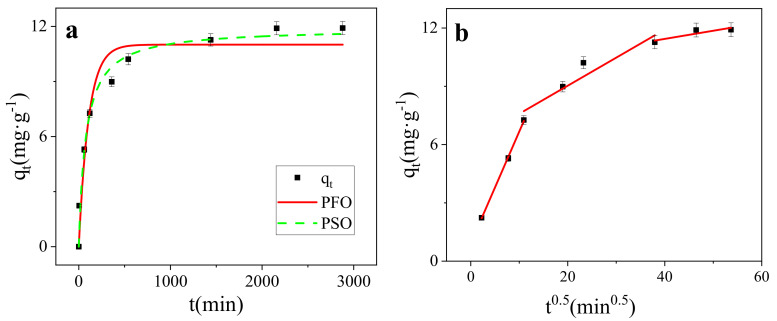
(**a**) Kinetics models of SMB (298 K); (**b**) Intraparticle diffusion model of SMB (298 K).

Adsorption is a time-dependent process [41]. As for the adsorption of MB on SMB, the adsorption process could be divided into two parts: fast adsorption stage and slow adsorption stage (Figure 6a). In the fast adsorption stage, the adsorption removal rate reached 85.74% of the entire removal efficiency, and then the adsorption rate decreased rapidly, and reached the adsorption equilibrium and the process of adsorption lasted for 2160 min. The change in adsorption rate may be due to the fact that all adsorption sites were initially empty and the concentration of MB was very high. The high concentration of MB formed a strong ion driving, which caused MB molecules to approach the surface of the adsorbent. Therefore, the adsorption rate was very fast in the initial stage. As the adsorption progressed, the adsorption sites on the adsorbent were occupied and the dye concentration decreased, so the adsorption rate also decreased [46,47].

In order to evaluate the adsorption mechanism of MB dye, pseudo-first-order (PFO) (Equation (1)), and pseudo-second-order (PSO) (Equation (2)) were used to fit the adsorption process.

(1) $$q_{t}=q_{e}\left(1-\exp^{-k_{1}t}\right)$$

(2) $$q_{t}=\frac{q_{e}^{2}k_{2}t}{1+q_{e}k_{2}t}$$

where *q_t_* (mg/g) and *q_e_* (mg/g) are the adsorption capacity at time t and at equilibrium respectively, *k*_1_ is the first order rate constant, *k*_2_ is the second order rate constant. The results were listed in Table 2.

The result showed that both the PFO and PSO can describe the adsorption kinetic data well (r^2^ > 0.90). However, compared with the PFO (r^2^ = 0.9454), the PSO (r^2^ = 0.9784) was more suitable in describing the adsorption kinetics of MB on SMB. This means that the main determinant of the predominantly adsorption rate of MB on SMB was chemical adsorption [48], which involves valency forces by the sharing or exchange of electrons between SMB and MB [49,50].

Dye adsorption in an aqueous solution usually includes the following steps: diffusing to the outer surface of the adsorbent through the solution (film diffusion); being adsorbed on the outer surface of adsorbent; diffusing from the outer surface of adsorbent to the internal of adsorbent (particle internal diffusion), and adsorption to the active center of the inner surface of adsorbent. The control step of the adsorption rate is usually membrane diffusion or ion diffusion [51]. In order to further explore the control step in the adsorption process, the intra-particle diffusion model (Equation (3)) was also used for fitting.

(3) $$q_{t}=k_{3}t^{0.5}+C$$

*k*_3_ is the rate constant of particle internal diffusion model, *C* (mg·g^−1^) represents the thickness of the boundary layer.

**Figure 7:**
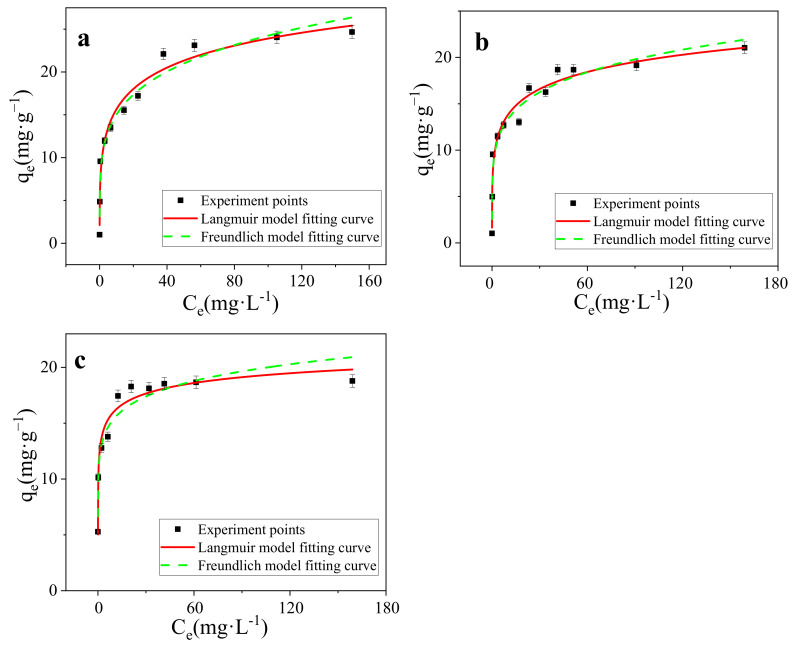
(**a**) Isotherm models of SMB (298 K), (**b**) Isotherm models of SMB (308 K), (**c**) Isotherm models of SMB (318 K).

It could be seen from Figure 7b that the fitted curve did not pass through the origin, which meant that the intra-particle diffusion was not the rate determining step of the adsorption mechanism of MB on SMB [52]. Adsorption is a multi-step process involving adsorption on the outer surface and diffusion in the interior [53], the entire MB adsorption process was divided into three stages. After MB molecules were adsorbed to the surface of SMB by molecular diffusion and membrane diffusion control, under the action of intra-particle diffusion, MB molecules entered the internal pore structure of SMB, and finally reached the adsorption center gradually to achieve adsorption equilibrium. In addition, as shown in Figure 6b, in the initial stage of adsorption, the slope is the largest and the intercept is the smallest, which indicates that the adsorption in this stage occurred very quickly. At this time, there were many remaining adsorption sites over the adsorbent, and the high MB concentration produced a strong ion driving force, so the adsorption in this stage was completed quickly. In the second stage, the adsorption rate was slower. In the last stage, the slope was the smallest and the intercept was the largest. At this stage, the adsorption site was gradually completely occupied by MB molecules, and the reaction reached the final equilibrium stage [34]. The adsorption of TC on magnetic biochar studied by Lin et al. also went through a similar process [41].

### 3.3. Isotherms Modeling

The adsorption isotherm is the definition of the relationship between the amount of adsorbate adsorbed per unit mass of adsorbent and adsorbate concentration in solution phase at constant temperature and at equilibrium condition [37]. As shown in Figure 7a–c), the sorption isotherm of MB onto SMB was “L” shape. The equilibrium adsorption capacity of MB on SMB increased with the increase of the initial MB concentration, similar findings have been found in previous studies [34,50]. In order to find the most suitable adsorption model, data was fitted to Langmuir (Equation (4)) and Freundlich (Equation (5)) sotherm models. The Langmuir isotherm can also be expressed by R_L_ (separation factor), the formula is shown in Equation (6) [54].

(4) $$q_{e}=\frac{k_{L}q_{m}C_{e}}{1+k_{L}C_{e}}$$

(5) qe=kFce1n

(6) $$R_{L}=\frac{1}{1+K_{L}C_{0}}$$

where *q_e_* (mg·g^−1^) is the amount of MB adsorbed per unit mass of the adsorbent when equilibrium is reached, *q_m_* (mg·g^−1^) is a theoretical maximum adsorption capacity. *Ce* (mg·L^−1^) is the MB concentration at equilibrium in the solution. The *K_L_* and *K_F_* are Langmuir and Freundlich constants respectively, and 1/n is the Freundlich index coefficient. *C*_0_ is the initial concentration. Parameters of these adsorption isotherms were presented in Table 2.

Both Langmuir and Freundlich models were applied to simulate the sorption isotherms (Figure 7a–c). All models reproduced the isotherm data well with a correlation coefficient (r^2^) above 0.90 (Table 2). For the Langmuir model, 0.9634 < r^2^ < 0.9749, shows a better description than the Freundlich model. Langmuir model assumed that the adsorption was a single layer adsorption, with the same limited adsorption sites on the homogeneous surface, suggesting that the chemical adsorption might play an important role in the adsorption of MB on SMB. At 298 K, adsorption advantage *R_L_* = 0.173, adsorption strength *n* = 0.208, *n* and *R_L_* are measures of adsorption preference, For this experiment, *n* < 1, *R_L_* < 1, proved that the adsorption of MB on SMB is a chemical adsorption process and SMB has the ability to adsorpt MB [55,56,57]. Freundlich adsorption isotherm is an empirical model, which is suitable for heterogeneous mass transfer systems and multilayer adsorption [58]. In this study, the Freundlich model also shows a high linear relationship (0.9316 < r^2^< 0.9666), suggesting that the adsorption of MB on SMB may also be closely related to physical interactions. 1/*n* is a parameter of Freundlich model, which can represent adsorption characteristics. The values of 1/*n* in this study are all less than 1, indicating that adsorption is both convenient and favorable. Great linear fitting based on two typical models (r^2^ was basically greater than 0.95), indicating that both chemical adsorption and physical adsorption may have played a role in the adsorption process, and the adsorption of MB onto SBM was mainly controlled by the Langmuir surface adsorption.

As shown in Table 2, the adsorption capacity of SMB at 25 °C, 35 °C, and 45 °C was 44.47 mg·g^−1^, 39.35 mg·g^−1^, and 25.85 mg·g^−1^ (the maximum adsorption capacities were calculated by Langmuir model), respectively. With the increase of temperature, the adsorption of MB on SMB showed a trend of decline, indicating that the reaction was exothermic, low temperature was conducive to the adsorption.

### 3.4. Effect of Initial Solution pH

**Figure 8:**
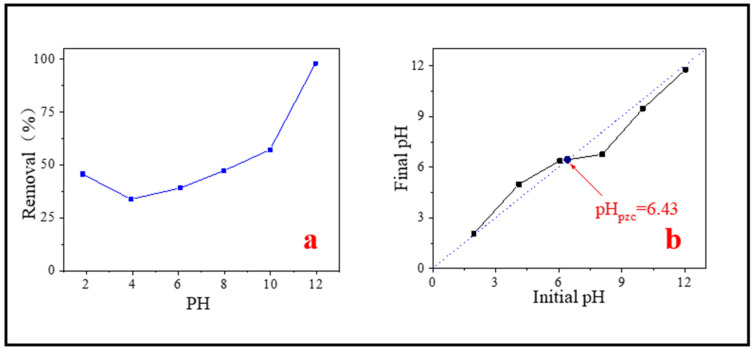
(**a**) Effect of initial solution pH on MB removal; (**b**) Determination of isoelectric point.

The pH of the solution is one of the most critical factors affecting the adsorption of pollutants in water [47]. Due to the different types of pollutants and the properties of adsorbents, the optimal pH for adsorbing pollutants also changes. Too high or too low pH will affect the adsorption effect. Therefore, the effect of initial solution pH on the removal of MB was studied. It could be seen from Figure 8a, as pH increased from 2 to 12, the removal capacity of SMB to MB first decreased and then increased. When pH was 4, the removal capacity was the worst. When pH was about 12, the adsorption effect was the best. There was a relative lyminor shift on MB removal efficiency under the condition of the pH range 4–8, only rose from 33.78% to 47.29%, but a sudden jump occurred in the range of 8–12, the removal efficiency rose from 47.29% to 97.97%. The adsorption experiments under different pH proved that the effect of pH on MB adsorption is not negligible, which may be related to the properties of MB molecule and the isoelectric point of the adsorbent. The research of Nhamo et al. also confirmed this [50].

According to the measurement, the isoelectric point of SMB was about 6.43 (Figure 8b). When the pH < pH_pzc_, the surface of SMB was positively charged. As the pH increases from 3.93 to 6.43, the repulsive force between SMB and MB (a kind of cationic adsorbent) slowly decreased and the adsorption capacity slowly increased. When the pH > pH_pzc_, the surface of the adsorbent was electronegative, and electrostatic attraction occurred between MB molecules and SMB. Therefore, when pH > 6.43, the removal efficiency of MB ions had been significantly improved. In addition, with the further increase of pH, the electrostatic adsorption effect was more intense, and the removal ability of SMB to MB was further improved. According to the experimental results, it is concluded that alkaline conditions are most conducive to adsorption. Other researchers have reported similar findings [59].

It is worth noting that this experiment did not reach the minimum adsorption value at the lowest pH. When pH is 1.84, the adsorption efficiency was 45.65%, which was greater than the adsorption efficiency when pH is 3.93. This may be because the BET of SMB was significantly increased after strong acid treatment (described in detail in the regeneration experiment), which was conducive to the adsorption, it made the adsorption capacity a little increase.

## 4. Reproducibility and Reusability

**Figure 9:**
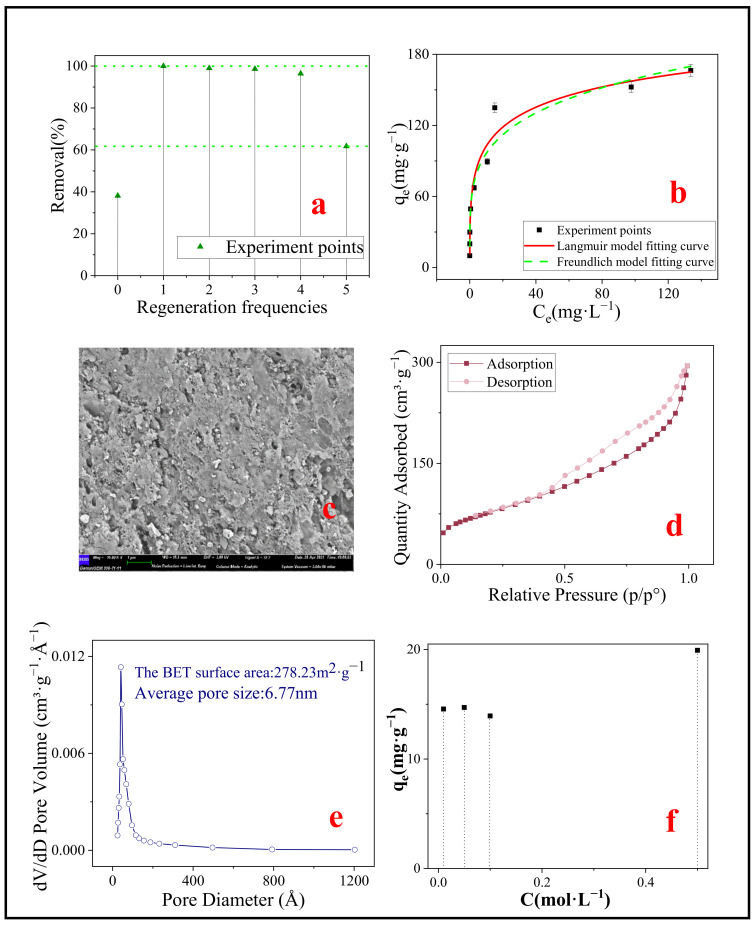
(**a**) Regeneration of SMB; (**b**) Isotherm models of SMB after one regeneration (298 K); (**c**) SEM image of SMB after one regeneration; (**d**) N_2_ adsorption–desorption isotherm liner plots of SMB after one regeneration; (**e**) BJH pore size distribution of SMB after one regeneration; (**f**) Adsorption capacity of SMB after one regeneration of hydrochloric acid at different concentrations.

In order to investigate the possibility of regeneration of used adsorbent, after the adsorption was completed, the desorption study was carried out using hydrochloric acid of different molar concentrations as the desorbent. Adsorption capacity of SMB after one regeneration of hydrochloric acid at different concentrations was shown in Figure 9f. The optimal regeneration concentration of 0.5 mol·L^−1^ was selected to regenerate the adsorbent. In addition, the adsorption after regeneration is shown in Figure 9a.

It could be noticed that the adsorption efficiency had been improved after regeneration (shown in Figure 9a). In the first three regenerations, the adsorption efficiency was almost 100%, and after the fifth regeneration, the adsorption efficiency still reached 61.73%, which was significantly higher than the adsorption rate of SMB without regeneration (38.14%). It is suspected that SMB may be modified by hydrochloric acid treatment, so the adsorption isotherm experiment was carried out on the SMB after one regeneration to investigate its adsorption capacity. The results of the isotherm model are shown in Figure 9b, and the theoretical maximum adsorption capacity fitted by the Langmuir model reached 296.52 mg·g^−1^. In addition, SEM (Figure 9c) and BET (Figure 9d,e) were used for characterization. It is worth mentioning that the adsorption capacity of SMB after one regeneration to MB was significantly higher than that of sludge-based biochar previously reported.

Previous studies have shown that exposure of biochar to an acidic solution can remove mineral elements, organic matter, and carbonate on the surface of biochar, and increase the number of micropore, which increases the roughness of the surface of biochar [60,61]. Comparing the SEM images of SMB (Figure 3d) and SMB after one regeneration (Figure 9c), it could be noticed that the surface roughness of SMB after one regeneration increased and more micropore were clearly visible. This was also confirmed in the BET results (Figure 9d,e). Compared with SMB, the surface area of SMB after one regeneration had increased from 20.19 m^2^·g^−1^ to 278.23 m^2^·g^−1^, the total pore volume had increased from 0.0819 cm^3^·g^−1^ to 0.1126 cm^3^·g^−1^, and the average pore diameter had decreased from 13.01 nm to 6.77 nm. The reason for this result may be that the modification process removed or dissolved the ash in the SMB, opened blocked pores of SMB, thus exposed more micropore inside the SMB [62]. Wang et al. obtained similar results by modifying rice husk biochar with nitric acid [63]. The increased surface area provides more attachment points for MB, which also explains why the adsorption effect of SMB after one regeneration has been greatly improved.

## 5. Conclusions

In this study, pyrolysis method was used to prepare magnetic biochar using backwashing iron mud from waterworks and sewage sludge, no chemicals were added during the whole reaction process. The results of this study are as follows: (1) This magnetic biochar could be used to remove MB molecules in the solution with the maximum adsorption capacity as high as 47.44 mg·g^−1^, and after 0.5 mol·L^−1^ hydrochloric acid modification, the adsorption capacity of the magnetic biochar for MB could be increased to 296.52 mg·g^−1^; (2) pH had a significant influence on the adsorption effect, SMB had the worst adsorption capacity when pH = 4, and the best adsorption capacity when pH = 12; (3) The magnetic material Fe_3_O_4_ in the product was well combined with biochar, and the saturation magnetization was 25.60 emu·g^−1^; (4) The adsorption process was more consistent with the Langmuir adsorption isotherm model and the pseudo-second-order model could better describe the adsorption kinetics of the adsorbent; (5) Even after five regenerations, the removal efficiency of MB could still reach 61.73%. In brief, this study provides a method for the preparation of a low-cost and effective adsorbent for the treatment of dyeing wastewater. 

## Figures and Tables

**Table 1:** Elemental composition of BC and SMB.

	N	C	H	Fe	Si	Na	K	Mg	Ca	P
	%, mass									
BC	2.48	12.65	0.88	3.94	0.66	1.73	1.77	1.78	4.43	8.72
SMB	2.50	18.94	0.66	14.25	0.16	0.94	1.30	1.30	1.60	4.66

**Table 2:** Parameters of the Kinetic model and Langmuir and Freundlich isotherm model parameters values for the adsorption of MB.

Model	Parameter 1	Parameter 2	r^2^
PFO	*k*_1_ = 0.009 min^−^^1^	*q_e_* = 11.01 mg·g^−^^1^	0.9454
PSO	*k*_2_ = 0.001 g·mg^−^^1^·min^−^^1^	*q_e_* = 11.90 mg·g^−^^1^	0.9784
Langmuir (298 K)	*K_L_* = 0.239 L·mg^−^^1^	*q_m_* = 47.44 mg·g^−^^1^	0.9749
	*R_L_* = 0.173	*C*_0_ = 20 mg·L^−^^1^	
Freundlich (298 K)	*K_F_* = 9.316 mg^(1−n)^·L^n^·g^−^^1^	1/*n* = 0.208	0.9666
Langmuir (308 K)	*K_L_* =0.288 L·mg^−^^1^	*q_m_* = 39.35 mg·g^−^^1^	0.9656
Freundlich (308 K)	*K_F_* =8.842 mg^(1−n)^·L^n^·g^−^^1^	1/*n* = 0.179	0.9584
Langmuir (318 K)	*K_L_* =0.916 L·mg^−^^1^	*q_m_* = 25.85 mg·g^−^^1^	0.9634
Freundlich (318 K)	*K_F_* =11.948 mg^(1−n)^·L^n^·g^−^^1^	1/*n* = 0.110	0.9316

## Supplementary Materials

The following are available online at https://www.mdpi.com/article/10.3390/nano11102473/s1, Figure S1: The main synthesis process of SMB.
