# Supplementary material for: Preparation and Characterization of Sludge-Based Magnetic Biochar by Pyrolysis for Methylene Blue Removal
Source: Nanomaterials (Basel). 2021 Sep 22;11(10):2473. doi: 10.3390/nano11102473 (PMC8539958; doi:10.3390/nano11102473)
Supplement: Supplementary file 1 [file nanomaterials-11-02473-s001.zip › nanomaterials-1381863-supplementary.pdf]

nanomaterials

Supplementary material

Preparation and Characterisation of Sludge Based Magnetic Biochar by Pyrolysis for Methylene Blue Removal

Huiping Zeng [1], Wei Qi [1], Longxue Zhai [1], Fanshuo Wang[2], Jie Zhang [1,3] and Dong Li [1,*]

1. Key Laboratory of Water Quality Science and Water Environment Recovery Engineering, civil engineering,Beijing University of Technology, Beijing 100124, China; zenghuiping@bjut.edu.cn (H.Z.); qw0529@163.com (W.Q.); zlx1017@163.com (L.Z.); 6282031@163.com (J.Z.)
2. Beijing Institute of Architectural Design, Beijing 100124, China; wfs960808@163.com (F.W.);
3. State Key Laboratory of Urban Water Resource and Environment, Harbin Institute of Technology, Harbin 150090, China

* Correspondence: lidongwerrc@163.com

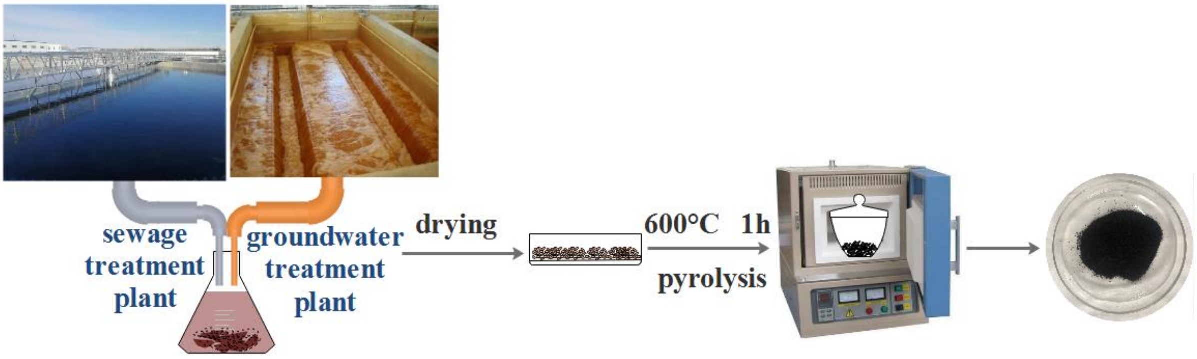

Scheme S1. The main synthesis process of SMB.
